# Blood Chromium Levels and Their Association with Cardiovascular Diseases, Diabetes, and Depression: National Health and Nutrition Examination Survey (NHANES) 2015–2016

**DOI:** 10.3390/nu14132687

**Published:** 2022-06-28

**Authors:** Jasmine Chen, Michael Kan, Pulindu Ratnasekera, Lovepreet Kaur Deol, Vidhi Thakkar, Karen M. Davison

**Affiliations:** 1 Health Science, Kwantlen Polytechnic University, Richmond, BC V6X 3X8, Canada; jasmine.chen@kpu.ca (J.C.); michael.kan@email.kpu.ca (M.K.); pulindu_ratnasekera@sfu.ca (P.R.); lovepreet.deol1@kpu.ca (L.K.D.); vidhithakkar@uvic.ca (V.T.); 2 Department of Statistics and Actuarial Science, Simon Fraser University, Burnaby, BC V5A 1S6, Canada; 3 Institute of Lifelong Aging and Health, University of Victoria, Victoria, BC V8P 5C2, Canada

**Keywords:** blood chromium, cardiovascular diseases, diabetes mellitus, depression, determinants of health

## Abstract

Currently, there is no global consensus about the essentiality of dietary chromium. To provide evidence to this debate, an examination of blood chromium levels and common chronic health conditions was undertaken. Using a subsample from the 2015–2016 US National Health and Nutrition Examination Survey (*n* = 2894; 40 years^+^), chi-square and binary logistic regression analyses were conducted to examine blood chromium levels (0.7–28.0 vs. <0.7 µg/L) and their associations with cardiovascular diseases (CVDs; self-report), diabetes mellitus (DM; glycohemoglobin ≥5.7%), and depression (Patient Health Questionnaire-9 score ≥5), while controlling for socio-demographic (age/sex/income/education/relationship status) and health-related (red blood cell folate/medications/co-morbidities/body mass index (BMI)/substance use) factors. The sample was almost evenly distributed between men and women (*n* = 1391, 48.1% (men); *n* = 1503, 51.9% (women)). The prevalence estimates of low blood chromium levels tended to be higher among those with CVDs (47.4–47.6%) and DM (50.0–51.6%). Comparisons between those with low vs. normal blood chromium levels indicate men have increased odds of CVDs (adjusted odds ratio (aOR) = 1.86, 95% confidence interval (CI): 1.22–2.85, *p* < 0.001) and DM (aOR = 1.93, 95% CI: 1.32–2.83, *p* < 0.001) and lower odds of depression (aOR = 0.42, 95% CI: 0.22–0.77, *p* < 0.05). Dietary chromium may be important in the prevention and management of CVDs and DM for men. Continued exploration of chromium’s role in chronic diseases, including differences by biological factors, is needed.

## 1. Introduction

Chromium is a trace dietary mineral that facilitates carbohydrate and lipid metabolism [1]. It is involved in glucose homeostasis as a critical cofactor for insulin action and as a component of the glucose tolerance factor [2]. Food sources of chromium include whole grain products, fruits, vegetables, nuts, bread, and meats [3,4].

About 60 years ago, chromium was deemed to be an essential dietary mineral based on clinical studies where deficiency symptoms were observed among those receiving total parenteral nutrition without chromium included in the solution [5,6]. However, in recent years, there has been debate about whether dietary chromium is an essential nutrient. The European Food Safety Authority Panel on Dietetic Products, Nutrition and Allergies has indicated that the data regarding chromium is too limited to establish recommended chromium intake levels [5,7] due to challenges in quantifying chromium in foods as well as body tissues and fluids [8,9,10]. Furthermore, most biological research examining suboptimal chromium intake and health outcomes has been limited to animal studies of chromium deficiency [7]. In the United States (US), Canada, Australia, and New Zealand population-based recommended intake levels have been established for chromium [11]. For example, The Institute of Medicine, which defines the US and Canada’s *Dietary Reference Intakes*, have established Adequate Intake (AI) levels for chromium as 35 µg/day for men and 25 µg/day for women between 19–50 years, and 30 µg/day for men and 20 µg/day for women 51 years^+^ [8]. Due to limited mechanistic and epidemiological studies available, no global consensus has been reached about the essentiality of dietary chromium [5]. 

Chromium has been linked with chronic conditions such as cardiovascular diseases (CVDs), diabetes mellitus (DM), and depression [12,13,14,15,16,17]. Chromium deficiency has been associated with DM and CVDs, such as atherosclerosis [18], and chromium supplementation has been reported to have beneficial effects on symptoms related to CVDs, type 2 DM, and depression [19,20]. It is also well known that these chronic physical and mental health conditions can result from complex interactions of socio-demographic, behavioral, environmental, psychological, and biological factors which can be catalyzed by challenging life events [21,22,23,24,25,26,27,28]. 

CVDs, DM, and depression impose significant global economic burdens. CVDs such as coronary heart disease, cerebrovascular disease, and rheumatic heart disease affect 523 million individuals worldwide [29] and account for 32.0% of all deaths globally [30]. The global incidence for DM is estimated at 22.9 million [31] and it is the one of the leading global causes of mortality [31,32]. Depression is a common mental health condition affecting 3.8% of the global population, including 5.0% among adults and 5.7% among adults older than 60 years [21,33]. It is the leading cause of disability worldwide [22].

Most of the research to date about dietary chromium and chronic health conditions has focused on the relationships between this trace mineral and DM. Cross-sectional and case-control studies have reported that individuals with DM have lower serum chromium levels compared to healthy individuals [2,34,35,36,37]. One cross-sectional study reported that serum chromium levels were significantly reduced in individuals with type 2 DM between 40 to 55 years compared to age and body mass index (BMI) matched controls [38]. A case-control study reported that plasma chromium levels were significantly lower in the type 2 and pre-DM groups compared to healthy individuals [39]. Furthermore, plasma chromium concentrations were inversely associated with type 2 DM and pre-DM in Chinese adults, and individuals with higher plasma chromium levels were less likely to have type 2 DM and pre-DM [39]. Some studies, including a systematic review and meta-analysis, have reported that supplementation of chromium can potentially help to control DM [20,36,40] by improving glycemic control [41]. 

Chromium may also be important in the prevention and management of CVDs. It has been shown that individuals with existing CVDs (e.g., coronary artery disease, myocardial infarction) have lowered plasma chromium levels compared to healthy individuals [42,43]. Plasma chromium levels have also been reported to be negatively correlated with blood pressure and low-density lipoproteins [44]. However, another study reported that individuals with systolic dysfunction had increased serum chromium levels compared to those with normal systolic function [45]. A cross-sectional study reported that increasing circulating levels of chromium is associated with reduced lipid infiltration in the vascular wall (echogenicity of the intima-media complex) in the elderly [46]. Systematic reviews and meta-analyses indicate that chromium supplementation may have protective effects against myocardial infarction, reduce CVD-associated inflammatory biomarkers [24], and decrease diastolic blood pressure [41]. 

There is also a limited body of research which has explored relationships between dietary chromium and depression [47]. Small clinical studies have reported that depression symptoms can be improved with chromium supplementation [19,48,49,50,51]. Interventions aimed at increasing chromium intakes have also been recommended adjuncts to anti-depressant medication treatment [52]. Clinical studies have reported that chromium supplementation improves glucose regulation, reduces appetite, decreased weight gain, and improves mood in individuals with depression [53].

Although research suggests that chromium may be important in the prevention and management of CVDs, DM, and depression, most of these studies are based on intakes from dietary or supplement sources. There has been limited investigations which have examined more objective measures of chromium status, such as blood levels of the mineral, in relation to these chronic physical and mental health conditions. Furthermore, most studies have not accounted for various other factors which can affect chromium status in their analyses. In the 2015–2016 US National Health and Nutrition Examination Survey (NHANES), blood levels of chromium were measured in participants 40 years^+^ and information about CVDs, DM, and depression was collected. The availability of this data provided an opportunity to examine relationships between chromium status with these common physical and mental health conditions, while controlling for various determinants of health. The findings of this investigation can contribute evidence which will either support or refute that dietary chromium is an essential trace mineral. It was hypothesized that there will be significant association between low blood levels of chromium and the presence of CVDs, DM, and depression.

## 2. Materials and Methods

### 2.1. Sample

An analysis of 2015–2016 US NHANES [54] data was conducted. The sample is comprised of a randomly selected non-institutionalized civilian population of all ages that reside in 50 states and the District of Columbia [55]. The stratified, clustered sampling was conducted in four stages: primary sampling units (PSUs) from all US counties (counties, groups of tracts within counties, or combinations of adjacent counties); segments within PSUs (census blocks or combinations of blocks); dwelling units (DUs); or households, within segments, and individuals within households [55]. Prior to data collection, screening was conducted at the DU level to identify eligible sampled persons based on oversampling subgroups, which included Hispanic persons, non-Hispanic black persons, non-Hispanic non-black Asian persons, non-Hispanic white persons and persons of other races and ethnicities at or below 185% of the poverty threshold, and non-Hispanic white persons and persons of other races and ethnicities aged 80 years^+^ [55]. The data collection consisted of in-home interviews using Computer-Assisted Personal Interviewing and physical examination at Mobile Examination Centers (MEC). In-home interviews included socio-demographic, dietary, and health-related questions. At the MEC, anthropometric, blood pressure, blood, and urine measures, hearing and vision tests, as well as health status assessments were conducted. Consent forms were signed by participants before in-home interviews and the MEC exams were conducted.

### 2.2. Measures

#### 2.2.1. Dependent Variables: Select Chronic Physical and Mental Health Conditions

This investigation included analysis of blood chromium levels in relation to three separate dependent variables: CVDs, DM, and depression. CVDs were coded as 0 (no) and 1 (yes) and were based on responses from questions about health condition diagnoses during the home-based interviews. CVDs included congestive heart failure, coronary heart disease, angina/angina pectoris, heart attack, stroke, and hypertension. 

The measure, DM, was based on glycohemoglobin levels obtained from a blood sample taken from participants in the MEC. The cut-off values used were <5.7% (no prediabetes or diabetes; code 0) and ≥5.7% (prediabetes or diabetes; code 1) [56]. Full details about blood sample collection and analysis are available elsewhere [57,58].

Depression was assessed using the Patient Health Questionnaire-9 (PHQ-9) [59,60]. The PHQ-9 is a nine-item depression screening instrument which incorporates Diagnostic and Statistical Manual of Mental Disorders fourth edition (DSM-IV) diagnostic criteria [61] to determine the frequency of depression symptoms over the past 2 weeks. The PHQ-9 was administered by trained interviewers in the MEC using the Computer-Assisted Personal Interview system which has built-in consistency checks [54]. When responding to individual questions, participants answered according to one of the following categories: “not at all”, “several days”, “more than half the days”, and “nearly every day”. Each category was assigned points ranging from 0 to 3. The sum of the score from the nine items indicated the level of depression severity. A score of 0 to 4, 5 to 9, 10 to 14, 15 to 19, and 20 to 27 indicated minimal, mild, moderate, moderately severe, and severe depression respectively [59,60]. A cut-off score of ≥5 was used for this analysis [59,60]. Based on studies in primary care settings, the PHQ-9 has shown good reliability and validity. Internal consistency of the PHQ-9 has been reported to have Cronbach alphas of 0.86 and 0.89, and criteria validity has been verified in a sample of 580 individuals where structured clinical interviews were conducted by a mental health professional [60].

#### 2.2.2. Independent Variable: Blood Chromium Levels

The main independent variable of interest was blood chromium levels. In the 2015–2016 US NHANES, human whole blood (optimal 2.0 mL, minimum 0.60 mL) samples were collected from participants (40 years^+^) in the MEC [62]. Full details of sample analysis, solutions preparation, base blood, and quality controls can be found in the Centers for Disease Control and Prevention (CDC) Laboratory Procedure Manual [62]. The maximum concentration of chromium that was measurable was 5000 µg/L and the lower limit of detection (LLOD) was 0.41 µg/L [62]. For analytes with results below the LLOD, an imputed fill value, calculated as the LLOD divided by the square root of 2 (LLOD/sqrt [2]), was used [62]. For this study, the healthy reference range of 0.7 to 28.0 µg/L for blood chromium levels was used [62,63,64,65]. The participants who provided blood samples also completed a questionnaire about fasting status, which collected data related to length of “food” fasts and whether the participant had gum, mints, coffee, tea, alcohol, or dietary supplements before their laboratory examination [62].

### 2.3. Covariates

The covariates included social, economic, demographic, biological, and health variables that can potentially alter the relationship between blood chromium levels and the studied chronic health conditions. Details of each variable are provided in the supplementary file (Supplementary Materials Table S1: Description of covariates). Further details about each variable are also located on the NHANES website [54].

### 2.4. Analysis

The demographics, examination, laboratory, and questionnaire datasets from the 2015–2016 cycle of the NHANES were merged using Statistical Package for the Social Sciences (SPSS) (version 24, IBM SPSS Statistics, Chicago, IL, USA) [66]. Descriptive analysis included frequency counts and percentages by categories for all variables. Pearson chi-square (χ^2^) tests were used on the unweighted data to assess for the associations between each of the three chronic conditions, blood chromium levels categories (low versus normal), and the covariates.

Three separate binary logistic regression analyses were conducted on the weighted data; each assessed the relationships between a given physical (CVDs, DM) or mental health (depression) condition while statistically controlling for all covariates. Adjusted odds ratios (aORs), 95% confidence intervals (CI), and *p*-values (level of significance < 0.05) were reported. The standardized weights were calculated by dividing the trimmed inflation weight of each unit used in the analysis by the unweighted average of the survey weights of all the analyzed units.

## 3. Results

### 3.1. Description of Overall Sample

The 2015–2016 NHANES sample of adults aged 40 years^+^ (*n* = 2894) were almost evenly distributed by men (*n* = 1391; 48.1%) and women (*n* = 1503; 51.9%). Most had blood chromium levels below the normal range (*n* = 2654, 91.7%) and none were above the reference range. Most were 60 years^+^ (*n* = 1443, 49.9%), had graduated from high school and/or have some post-secondary education (*n* = 1461, 50.5%), were married and/or living with a partner (*n* = 1828, 63.2%), and had an annual income less than $25,000 USD (*n* = 883, 30.5%) (Supplementary Materials Table S2). Most had red blood cell (RBC) folate levels below/within the normal range (*n* = 2034, 70.3%) and no health conditions (*n* = 1158, 40.0%). Most were considered obese (*n* = 1267, 43.8%). About half of the sample were taking medications used to treat CVDs (*n* = 1479, 51.1%).

### 3.2. Analysis by Chronic Physical or Mental Health Condition

#### 3.2.1. CVDs

About half of the men (*n* = 723, 52.0%) and women (*n* = 783, 52.1%) had CVDs and among those with these health conditions, almost half had low blood chromium levels (*n* = 660, 47.4% (men); *n* = 716, 47.6% (women)) (Supplementary Materials Table S2). Prevalence estimates of CVDs for men and women were higher among those aged 60 years^+^ (*n* = 466, 33.5% (men); *n* = 500, 33.3% (women)), high school graduates or those who had some post-secondary education (*n* = 400, 28.8% (men); *n* = 412, 27.4% (women)), those who were married and/or living with partner (*n* = 504, 36.2% (men); *n* = 378, 25.1% (women)), and those who earned less than $25,000 USD per year (*n* = 250, 18.0% (men); *n* = 311, 20.7% (women)) (Supplementary Materials Table S2). Men and women with RBC folate levels below/within the normal range (*n* = 475, 34.1% (men); *n* = 506, 33.7% (women)), with no depression (*n* = 555, 39.9% (men); *n* = 504, 33.5% (women)), or with DM ((*n* = 477, 34.3% (men); *n* = 529, 35.2% (women)) had among the highest prevalence estimates for CVDs. Men considered to be moderate drinkers (*n* = 301, 21.6%) or who had smoked at least 100 cigarettes in their lifetime (*n* = 472, 33.9%) had higher levels of CVDs. Unlike men, women who did not report drinking behavior (*n* = 396, 26.3%) and had smoked less than 100 cigarettes in their lifetime (*n* = 492, 32.7%) had higher prevalence estimates of CVDs.

Results of the χ^2^ statistical analysis indicate that blood chromium levels are not associated with CVDs. However, most socio-demographic and health-related factors were significantly associated with CVDs (*p*’s < 0.05) (Supplementary Materials Table S2). Specifically, associations with CVDs were found for age, education, marital status, income, RBC folate, multi-morbidity, DM, depression (women only), BMI, CVDs-related medications, drinking behavior (women only), and smoking.

Only men with low blood chromium levels had significantly higher odds of CVDs when compared to normal blood chromium levels (aOR = 1.86, 95% CI: 1.22–2.85, *p* < 0.001) (Table 1). In addition, significant associations were observed for socio-demographic and health-related variables among men and women. For women who were more educated, there were lower odds of CVDs compared to those who were not high school graduates (aOR = 1.75 vs. 2.20, *p*’s < 0.001). For men, the odds of CVDs were 2.36 times higher for those who were married and/or living with a partner compared to those who were widowed, divorced, or separated (aOR = 2.36, 95% CI: 1.38–4.03, *p* < 0.001). Among health-related measures, men with one health condition (aOR = 0.68, 95% CI: 0.50–0.93, *p* < 0.05) had lower odds of CVDs compared to those with no health conditions. Compared to individuals with weights in the healthy range, those who were overweight or obese had lower odds of depression (aOR = 0.44–0.63 (men), 0.43–0.56 (women), *p*’s < 0.001). For health behaviors, men who smoked at least 100 cigarettes in their lifetime had lower odds of CVDs (aOR = 0.76, 95% CI: 0.59–0.97, *p* < 0.05) compared to those who smoked less than 100 cigarettes. Women who were not moderate drinkers had higher odds of CVDs (aOR = 1.45, 95% CI: 1.02–2.96, *p* < 0.05).

#### 3.2.2. DM

In the total sample (*n* = 2894), about one quarter of both men (*n* = 783/2894, 27.1%) and women had DM (*n* = 829/2894, 28.6%) and, among those with this condition, at least 50% had low blood chromium levels (*n* = 718, 51.6% (men); *n* = 752, 50.0% (women)) (Supplementary Materials Table S2). Prevalence estimates of DM that were more than one quarter of the sample for men and women respectively were found among those 60 years^+^ (*n* = 459, 33.0% (men); *n* = 480, 31.9% (women)), who were high school graduates or had some post-secondary education (*n* = 393, 28.3% (men); *n* = 406, 27.0% (women)), were married and/or living with partner (*n* = 576, 41.4% (men); *n* = 426, 28.3% (women)), had RBC folate levels below/within the normal range (*n* = 551, 39.6% (men); *n* = 558, 37.1% (women)), had at least two health conditions (*n* = 410, 29.5% (men); *n* = 495, 32.9% (women)), or no depression (*n* = 602, 43.3% (men); *n* = 556, 37.0% (women)). Men who were considered obese (*n* = 348, 25.0%) or overweight (*n* = 296, 21.3%) had similar prevalence estimates for DM. Men who were considered moderate drinkers (*n* = 322, 23.1%) and had smoked at least 100 cigarettes in their lifetime (*n* = 474, 34.1%) had higher prevalence of DM. Women who did not report drinking behavior (*n* = 432, 28.7%) or smoked less than 100 cigarettes (*n* = 545, 36.3%) had higher prevalence of DM.

The results of the χ^2^ statistical analyses indicated that blood chromium levels were not significantly associated with DM. However, some socio-demographic factors and most health-related factors were significantly associated with DM (*p*’s < 0.05) (Supplementary Materials Table S2); specifically, age, education, marital status (women only), income, multi-morbidity, depression (women only), BMI, DM-related medications, and drinking behavior. 

The odds of DM were 1.93 times higher for men with low blood chromium levels compared to those with normal levels (aOR = 1.93, 95% CI: 1.32–2.83, *p* < 0.001) (Table 1). Men and women between 50–59 years had lower odds of DM (aOR 0.47–0.48, *p*’s < 0.001) compared to those between 40–49 years. For men who were high school graduates or had some post-secondary education, the odds of DM were higher (aOR = 1.55, 95% CI: 1.04–2.31, *p* < 0.05) compared to those who were not high school graduates. Women who were at least post-secondary graduates had lower odds of DM (aOR = 0.64, 95% CI: 0.50–0.81, *p* < 0.001). Men who were married or living with a partner (aOR = 3.22, 95% CI: 1.87–5.53, *p* < 0.001) or never married (aOR = 3.50, 95% CI: 2.11–5.79, *p* < 001) had higher odds of DM when compared to those who were separated, divorced, or widowed. Among health-related measures, men and women with high RBC folate levels had higher odds of DM (aOR = 1.55 (men), aOR = 1.33 (women), *p*’s < 0.05) compared to those with RBC folate levels below/within the normal ranges. Men who were diagnosed with two or more health conditions had higher odds of DM (aOR = 1.36, 95% CI: 1.02–1.83, *p* < 0.05) compared to those who had no health conditions. Men and women who reported depression had lower odds of DM (aOR = 0.69 (men), 0.62 (women), *p*’s < 0.05) compared to those who did report depression. Men and women who were overweight or obese had lower odds of DM (aORs range 0.28–0.55, *p*’s < 0.001) compared to those whose body weight were within the healthy range. For health behaviors, men and women who did not report alcohol drinking had lower odds of DM (aOR = 0.64 (men), aOR = 0.51 (women), *p*’s < 0.05) compared to those who reported moderate levels of drinking. Men who smoked at least 100 cigarettes in their lifetime had lower odds of DM (aOR = 0.70, 95% CI: 0.55–0.89, *p* < 0.05) compared to those who smoked less than 100 cigarettes.

#### 3.2.3. Depression

Less than one quarter of men (*n* = 296, 21.3%) and almost one-third of women (*n* = 445, 29.6%) had depression. Among those with this condition, about one quarter had low blood chromium levels (*n* = 273, 19.6% (men); *n* = 409, 27.2% (women)) (Supplementary Materials Table S2). The prevalence of depression for both men and women was higher among those who were 60 years^+^ (*n* = 145, 10.4% (men); *n* = 213, 14.2% (women)), high school graduates or those who had some post-secondary education (*n* = 142, 10.2% (men); *n* = 229, 15.2% (women)), married and/or living with partner (*n* = 188, 13.5% (men); *n* = 220, 14.6% (women)), and earned less than $25,000 USD per year (*n* = 125, 9.0% (men); *n* = 191, 12.7% (women)). Men and women with RBC folate levels below/within the normal range (*n* = 210, 15.1% (men); *n* = 297, 19.8% (women), two or more health conditions (*n* = 181, 13.0% (men); *n* = 300, 20.0% (women)), or DM (*n* = 181, 13.0% (men); *n* = 273, 18.2% (women)) had higher prevalence estimates of depression. Men who drank moderate amounts of alcohol (*n* = 101, 7.3%) or smoked at least 100 cigarettes (*n* = 203, 14.6%) had higher prevalence of depression. Women who did not report drinking alcohol (*n* = 208, 13.8%) or smoked less than 100 cigarettes (*n* = 250, 16.6%) had higher prevalence estimates of depression.

Results of the χ^2^ statistical analysis indicated that blood chromium levels were not significantly associated with depression. However, most socio-demographic and health-related variables were significantly associated with depression (*p*’s < 0.05). Specifically, age (men only), education, marital status, income, multi-morbidity, DM (women only), BMI (women only), mental health-related medications, drinking behavior, and smoking were significantly associated with depression.

Men with low blood chromium levels had lower odds of depression (aOR = 0.42, 95% CI: 0.22–0.77, *p* < 0.05) compared to those with normal levels (Table 1). Similar to the results for CVDs and DM, significant associations were observed for socio-demographic and health-related variables. Men and women 50 years^+^ had higher odds of depression (aOR = 1.72–3.50, *p*’s < 0.05) compared to those between 40–49 years. Men and women who were high school graduates or had some post-secondary education as well as women who were post-secondary graduates had higher odds of depression (aORs = 1.40–1.93, *p*’s < 0.05) compared to those who were not high school graduates. The odds of depression for men who were married and/or living with a partner were almost three times higher (aOR = 2.86, 95% CI: 1.60–5.14, *p* < 0.001) compared to those who were widowed, divorced, or separated. Among health-related measures, men with high RBC folate levels had higher odds of depression (aOR = 1.51, 95% CI: 1.10–2.06, *p* < 0.05) compared to those with levels below/within the normal range. Individuals with at least one health condition had lower odds of depression (aORs = 0.38–0.61, *p*’s < 0.05) compared to those who had no health conditions. Women with DM had lower odds of depression (aOR = 0.54, 95% CI: 0.43–0.69, *p* < 0.001) compared to those without DM. Individuals taking mental health-related medications had lower odds of depression (aOR = 0.23 (men), 0.31 (women), *p*’s < 0.001) compared to those who did not take these medications.

## 4. Discussion

This study examined associations between blood chromium levels and CVDs, DM, and depression. Among adults aged 40 years^+^, the prevalence estimates of CVDs, DM, and depression are higher for men and women with low blood chromium levels compared to those with normal levels. Bivariate analysis indicated no significant associations between blood chromium levels and the three health outcomes. Results of the binary logistic regression analyses, where various determinants of health were accounted for, indicated significant associations between blood chromium levels and the three health outcomes. Notably, men with low blood chromium levels were more likely to have CVDs and DM and less likely to have depression.

The results found for men are consistent with existing knowledge that suggests inadequate dietary chromium is associated with CVDs and DM [12,13,14,15,16]. Furthermore, it supports the higher AI levels of dietary chromium the Institute of Medicine has set for mid-age and older men (19–50 years: AI 35 µg/day for men and 25 µg/day for women; 51 years^+^: 30 µg/day for men and 20 µg/day for women [8]). Studies indicate that trivalent chromium is a cofactor for a biologically active molecule that enhances the effects of insulin, a hormone which influences the metabolism of carbohydrates, fat, and protein [70], and therefore plays a role in CVDs and DM. Previous studies suggest a possible mechanism of action includes a chromium-induced increase in the insulin receptor number and insulin binding at its site of action [71]. Chromium increases 5′-AMP-activated protein kinase activity, resulting in the suppression of a sterol regulatory element-binding protein (SREBP)-1. SREBP-1 contributes to the synthesis and uptake of cholesterol, triglycerides, fatty acids, and phospholipids [12,72]. Chromium has been shown to increase free fatty acid oxidation and decrease fatty acid synthesis [20]. Among those with DM and CVDs, low blood levels of chromium are prevalent and this might be due to the diminution of insulin signal transduction and contribute to insulin resistance [73]. Chromium losses and excretion increase with aging and are also related to DM [73]. When losses occur among those with DM for more than 2 years, chromium homeostasis may be altered [74].

It is surprising that for women no significant associations between blood chromium levels and CVDs or DM were found. However, in a separate analysis, where the outcome of DM was based on self-report, a significant association for women was found (aOR = 1.99, *p* < 0.001). Although glycohemoglobin is an objective measure, it is important to note that two elevated results are needed for diagnosis of prediabetes or diabetes. The NHANES only reported one result. It is possible that a more accurate estimate of the prevalence of prediabetes and diabetes in the sample was somewhere between the indicated glycohemoglobin values and the self-report measure of DM. As such, there may be significant association between blood chromium levels and DM for women also.

The results related to blood chromium and depression seem counterintuitive. When compared to those with normal blood chromium levels, men had lower odds for depression and for women no significant association was found. It was thought this may be due to the PHQ-9 cut-off of mild depression which was used. However, when we conducted a subsequent analysis with a higher moderate depression cut-off (>9), the association between men and women with low blood chromium levels was non-significant. It has been reported that chromium functions in regulating fat metabolism and energy homeostasis by enhancing insulin sensitivity in the hypothalamus, resulting in increased production of serotonin, norepinephrine, and melatonin [52,53]. It has also been indicated that the serotonergic pathway and polymorphisms that occur in genes related to the pathway are strongly linked with depression [75]. Based on findings from pre-clinical and clinical studies, chromium may have antidepressant potential as it has been shown that the provision of supplemental sources improves glucose regulation and improves mood in individuals with depression [53]. However, many of these studies did not account for either dietary intake or blood levels of chromium or for other relevant factors such as RBC folate status, presence of physical health conditions, or substance use. Clearly, further investigative work related to chromium and depression is needed.

Consistent with other findings, our study reports that factors such as older age, education, relationship status, income, high RBC folate levels, multimorbidity, BMI, alcohol intake, and smoking are significantly associated with CVDs, DM, and depression [21,22,23,24,25,26,27,28,76]. However, there are some surprising results. Across the three conditions, older age showed both lower (CVDs in women; DM in men and women) and higher odds (depression in men and women). Differences in the odds of depression by educational attainment and income levels may be due to the lack of account of current employment status. Longitudinal investigations have indicated that, among those without depression at baseline, individuals with a secondary school education or less have a higher likelihood of developing a major depressive episode than more educated working respondents [77]. However, among the unemployed, a higher risk of major depression was found for those with a higher level of education [77]. The higher odds of CVDs among men who were married or living with a partner was surprising. Better prognosis in married individuals has been reported after myocardial infarction [78,79,80,81,82,83] and stroke [84], whereas other studies found that marital status had no influence on CVD [85,86,87]. The lower odds of depression among those with DM may be because many had their condition long-term, and they have adjusted psychologically. The lower odds of CVDs and depression found among co-morbidity levels may be because other common conditions with high morbidity (e.g., DM), were included as separate variables in the regression analysis. The lower odds of CVDs and DM among men and women who were overweight or obese may be a result of applying the same BMI cut-offs across all ages. Some studies suggest that BMI status and disease risk across adult age groups has a U-shaped relationship [88]. Finally, the lower odds of CVDs and DM among men who smoked more than 100 cigarettes in their lifetime may be due to measurement concerns with the 100-cigarette smoking screen [89].

To the best of our knowledge, this study appears to be among the first to report associations between blood chromium levels and common chronic physical and mental health conditions, while accounting for several determinants of health. The sex-specific significant associations found for CVDs and DM suggest that chromium has an essential role in the prevention and management of these conditions, and these differ among men and women. In industrial countries, insufficient intakes of dietary chromium have been reported and found to be associated with alterations in glucose metabolism, especially in older adults [90]. Other studies have also found associations of low plasma chromium levels with hyperglycemia, insulin resistance, high inflammatory status, and increased cardiovascular risk [44]. There is evidence for the efficacy of chromium supplementation to improve dyslipidemia and glucose levels in type 2 DM [91]. However, knowledge gaps remain in terms of how chromium deficiency and toxicity may be defined and who is affected. Future investigations could include subgroup analyses of chromium status and disease outcomes, where feasible, across different age and ethnic groups. Longitudinal analysis would also allow for causal inferences to be made about blood chromium levels and chronic condition outcomes. Studies which focus on improved measures of chromium intake and status would advance knowledge about the role of this trace mineral in chronic disease prevention and management.

The results of this study need to be considered in the context of its limitations. Given that the data was cross-sectional, the direction of the relationships between the health outcomes and blood chromium levels cannot be ascertained. There are many pathophysiological processes that are involved in CVDs, DM, and depression and thus it was impossible to account for all variables that may affect the relationships between the health outcomes and different health determinants. The use of self-report measures for many of the variables made misreporting and misclassification possible. Variables such as dietary chromium intake, ethnicity, other blood nutrient levels, dietary intakes, mental/brain-related health conditions, and illicit drug use were not included in the logistic regression analysis as they were unavailable. We used the reference ranges for blood chromium available from the CDC; however, it should be noted that there is no consensus on an international acceptable range in the general population. Finally, inferences made from our investigation only pertain to adults 40 years^+^ since the collection of blood chromium samples were only performed for this age group.

## 5. Conclusions

CVDs, DM, and depression are leading contributors to disability worldwide. This study, which examined relationships between blood chromium levels, CVDs, DM, and depression, while accounting for other important health determinants showed that men with low blood chromium levels had higher odds of CVDs and DM. These findings suggest chromium is essential for human health and its requirements in relation to these health outcomes differ between men and women. These results appear to indicate that chromium is an essential trace mineral which should be included in population-based dietary intake guidelines. Further investigations related to the role of chromium in physical and mental health conditions are warranted, particularly those which examine differences by sex and other biological variables such as age, ethnicity, and genetics.

## Figures and Tables

**Table 1:** Adjusted odds ratios (aOR with 95% confidence interval, *p*-value) of health conditions by demographic, social, economic, biological, and health variables.

Variable (Reference Category)	Cardiovascular Diseases		Diabetes Mellitus ^a^		Depression ^b^	
	Men	Women	Men	Women	Men	Women
**1. Blood Chromium Levels** (Within range of 0.7–28.0 µg/L)						
Below range (<0.7 µg/L)	1.86 (1.22–2.85, 0.00)	1.11 (0.71–1.73, 0.66)	1.93 (1.32–2.83, 0.00)	0.88 (0.63–1.22, 0.44)	0.42 (0.22–0.77, 0.01)	0.93 (0.65–1.33, 0.71)
**2. Socio-demographic Characteristics**						
**Age** (40–49 years)						
50–59 years	1.09 (0.77–1.54, 0.62)	0.66 (0.44–0.98, 0.04)	0.48 (0.35–0.66, 0.00)	0.47 (0.35–0.63, 0.00)	1.72 (1.15–2.57, 0.01)	2.44 (1.78–3.36, 0.00)
60 years^+^	1.20 (0.88–1.64, 0.24)	0.95 (0.67–1.34, 0.77)	1.11 (0.83–1.48, 0.48)	0.78 (0.60–1.01, 0.06)	3.50 (2.49–4.90, 0.00)	1.94 (1.47–2.56, 0.00)
**Education** (Less than high school graduate)						
High school graduate and/or some post–secondary education	0.90 (0.59–1.37, 0.63)	2.20 (1.32–3.64, 0.00)	1.55 (1.04–2.31, 0.03)	0.92 (0.63–1.34, 0.66)	1.74 (1.10–2.75, 0.02)	1.93 (1.30–2.86, 0.00)
Post–secondary graduate or above	1.62 (1.21–2.17, 0.00)	1.75 (1.25–2.45, 0.00)	1.18 (0.90–1.55, 0.22)	0.64 (0.50–0.81, 0.00)	1.15 (0.81–1.64, 0.43)	1.40 (1.06–1.86, 0.02)
**Marital Status** (Widowed/divorced/separated)						
Married/living with partner	2.36 (1.38–4.03, 0.00)	0.86 (0.50–1.50, 0.60)	3.22 (1.87–5.53, 0.00)	1.35 (0.87–2.10, 0.18)	2.86 (1.60–5.14, 0.00)	0.80 (0.52–1.23, 0.31)
Never married	1.30 (0.80–2.10, 0.29)	0.69 (0.41–1.18, 0.18)	3.50 (2.11–5.79, 0.00)	1.32 (0.86–2.02, 0.21)	1.58 (0.91–2.77, 0.11)	0.75 (0.49–1.14, 0.18)
**Family Income** ($75,000^+^ USD)						
$65,000–74,999 USD	0.32 (0.15–0.69, 0.00)	1.52 (0.66–3.54, 0.33)	0.69 (0.35–1.38, 0.29)	0.64 (0.33–1.25, 0.19)	0.86 (0.32–2.28, 0.76)	0.83 (0.40–1.71, 0.62)
$55,000–64,999 USD	0.16 (0.06–0.39, 0.00)	1.23 (0.45–3.35, 0.69)	0.63 (0.28–1.39, 0.25)	0.53 (0.24–1.15, 0.11)	1.12 (0.38–3.32, 0.84)	0.65 (0.27–1.57, 0.34)
$45,000–54,999 USD	0.32 (0.13–0.74, 0.01)	1.87 (0.73–4.78, 0.19)	0.69 (0.32–1.47, 0.33)	0.71 (0.33–1.49, 0.36)	1.53 (0.55–4.29, 0.41)	1.79 (0.81–3.92, 0.15)
$35,000–44,999 USD	0.63 (0.26–1.49, 0.29)	2.57 (1.03–6.41, 0.04)	1.89 (0.88–4.06, 0.10)	0.80 (0.39–1.64, 0.54)	1.97 (0.70–5.56, 0.20)	1.08 (0.50–2.35, 0.84)
$25,000–34,999 USD	0.31 (0.13–0.73, 0.01)	2.62 (1.04–6.62, 0.04)	1.37 (0.62–3.00, 0.43)	0.55 (0.26–1.13, 0.10)	1.10 (0.38–3.20, 0.86)	1.04 (0.47–2.29, 0.92)
Less than $25,000 USD	0.30 (0.13–0.72, 0.01)	1.83 (0.78–4.30, 0.17)	1.07 (0.50–2.31, 0.86)	0.96 (0.49–1.90, 0.91)	2.73 (0.99–7.51, 0.05)	1.47 (0.71–3.06, 0.30)
Not reported	0.50 (0.22–1.13, 0.10)	4.07 (1.75–9.44, 0.00)	1.33 (0.64–2.75, 0.44)	1.15 (0.59–2.25, 0.68)	1.95 (0.73–5.23, 0.18)	1.86 (0.92–3.78, 0.09)
**3. Biological Measurements**						
**Red Blood Cell Folate ^c^** (Below/within range: <317 or 317–1422 nmol/L)						
Above range (>1422 nmol/L)	0.80 (0.62–1.05, 0.11)	0.84 (0.63–1.12, 0.23)	1.55 (1.20–2.00, 0.00)	1.33 (1.07–1.66, 0.01)	1.51 (1.10–2.06, 0.01)	0.94 (0.75–1.19, 0.63)
**4. Health Measures**						
**Multi–morbidity ^d^** (No health conditions)						
One health condition	0.68 (0.50–0.93, 0.02)	0.80 (0.55–1.14, 0.22)	0.93 (0.69–1.26, 0.66)	0.78 (0.58–1.05, 0.10)	0.38 (0.26–0.56, 0.00)	0.35 (0.25–0.50, 0.00)
Two or more health conditions	0.84 (0.60–1.16, 0.29)	0.77 (0.55–1.08, 0.13)	1.36 (1.02–1.83, 0.04)	0.80 (0.62–1.04, 0.09)	0.61 (0.43–0.87, 0.01)	0.55 (0.41–0.73, 0.00)
**Diabetes ^a^** (No diabetes or prediabetes; glycohemoglobin <5.7%)						
Have diabetes or prediabetes	0.79 (0.61–1.01, 0.06)	0.77 (0.58–1.03, 0.08)	–	–	0.81 (0.61–1.07, 0.14)	0.54 (0.43–0.69, 0.00)
**Depression ^b^** (No depression)						
Yes/depressed	1.32 (0.95–1.83, 0.10)	0.74 (0.54–1.01, 0.06)	0.69 (0.51–0.93, 0.01)	0.62 (0.49–0.79, 0.00)	–	–
**Body Mass Index (BMI) ^e^** (Within healthy weight range)						
Overweight	0.44 (0.31–0.62, 0.00)	0.43 (0.30–0.61, 0.00)	0.47 (0.34–0.64, 0.00)	0.28 (0.22–0.37, 0.00)	1.00 (0.67–1.50, 0.98)	0.85 (0.64–1.14, 0.29)
Obese	0.63 (0.49–0.83, 0.00)	0.56 (0.40–0.78, 0.00)	0.55 (0.42–0.70, 0.00)	0.46 (0.36–0.59, 0.00)	1.32 (0.97–1.80, 0.08)	1.01 (0.78–1.32, 0.94)
**Cardiovascular Diseases-related Medication** (None)						
Take one or more cardiovascular diseases-related medication(s)	0.07 (0.05–0.09, 0.00)	0.03 (0.02–0.04, 0.00)	–	–	–	–
**Mental Health-related Medication ^f^** (None or not reported)						
Take one or more mental health-related medication(s)	–	–	–	–	0.23 (0.17–0.31, 0.00)	0.31 (0.25–0.39, 0.00)
**Diabetes Mellitus-related Medication** (None)						
Take one or more diabetes mellitus-related medication(s)	–	–	0.02 (0.01–0.04, 0.00)	0.08 (0.05–0.14, 0.00)	–	–
**5. Health Behavior Variables**						
**Drinking Behavior ^g^** (Moderate drinking)						
Not moderate drinking	0.83 (0.61–1.14, 0.25)	1.45 (1.02–2.06, 0.04)	1.10 (0.83–1.47, 0.51)	0.85 (0.66–1.11, 0.23)	0.87 (0.62–1.24, 0.45)	1.04 (0.78–1.38, 0.78)
Not reported	0.75 (0.53–1.06, 0.10)	1.32 (0.91–1.91, 0.15)	0.64 (0.47–0.89, 0.01)	0.51 (0.39–0.67, 0.00)	0.97 (0.67–1.41, 0.88)	1.39 (1.04–1.87, 0.03)
**Smoking** (<100 cigarettes in lifetime)						
≥100 cigarettes in lifetime	0.76 (0.59–0.97, 0.03)	1.02 (0.76–1.36, 0.92)	0.70 (0.55–0.89, 0.00)	1.22 (0.98–1.52, 0.08)	0.75 (0.56–1.01, 0.06)	0.87 (0.69–1.10, 0.24)

^a^ Diabetes and prediabetes: ≥5.7% glycohemoglobin [56]; ^b^ Depression: total Patient Health Questionnaire-9 (PHQ-9) score ≥ 5 [59,60]; ^c^ Clinical reference ranges of 317–1422 nmol/L [67]; ^d^ Includes cardiovascular diseases (congestive heart failure, coronary heart disease, angina/angina pectoris, heart attack, stroke, high blood pressure), respiratory diseases (emphysema, chronic bronchitis, chronic obstructive pulmonary disease (COPD), asthma), kidney diseases (weak/failing kidneys, kidney stones), thyroid disease, liver diseases, bone diseases (arthritis, gout), cancer. Note: cardiovascular diseases are excluded in the multi-morbidity variable when the dependent variable is cardiovascular diseases; ^e^ BMI: body mass index. Within healthy weight range: 18.5–24.9 kg/m^2^; overweight: 25–30 kg/m^2^; obese: ≥30 kg/m^2^ [68]; ^f^ Includes anti-depressant, psychiatric medication(s), or medication(s) for depression, feeling worried, or anxious; ^g^ Moderate drinking: ≤1 alcoholic drinks/day for women and ≤2 alcoholic drinks/day for men. Not moderate drinking: >1 alcoholic drinks/day for women and >2 alcoholic drinks/day for men [69]. 75,000^+^ USD means 75,000 and over 75,000 USD. 60 years^+^ means 60 and over 60 years old. $ means American currency.

## Data Availability

Data for this study is located at www.cdc.gov/nchs/nhanes (accessed on 30 May 2022).

## Supplementary Materials

The following supporting information can be downloaded at: https://www.mdpi.com/article/10.3390/nu14132687/s1, Table S1: Description of covariates, Table S2: Description of sample for health conditions.

## References

[1] Vincent J.B. (2010). Chromium: Celebrating 50 years as an essential element?. Dalton Trans..

[2] Siddiqui K., Bawazeer N., Joy S.S. (2014). Variation in macro and trace elements in progression of type 2 diabetes. Sci. World J..

[3] National Institutes of Health (NIH)—Office of Dietary Supplements Chromium—Fact Sheet for Health Professionals. https://ods.od.nih.gov/factsheets/Chromium-HealthProfessional/.

[4] Anderson R.A., Bryden N.A., Polansky M.M. (1992). Dietary chromium intake. Freely chosen diets, institutional diet, and individual foods. Biol. Trace Elem. Res..

[5] Vincent J.B. (2017). New evidence against chromium as an essential trace element. J. Nutr..

[6] Raymond J.L., Morrow K. (2021). Krause and Mahan’s Food and the Nutrition Care Process.

[7] European Food Safety Authority (EFSA) Panel on Dietetic Products, Nutrition and Allergies (NDA) (2014). Scientific opinion on dietary reference values for chromium. EFSA J..

[8] Institute of Medicine (US) Panel on Micronutrients (2001). Dietary Reference Intakes for Vitamin A, Vitamin K, Arsenic, Boron, Chromium, Copper, Iodine, Iron, Manganese, Molybdenum, Nickel, Silicon, Vanadium, and Zinc.

[9] Lukaski H.C. (1999). Chromium as a supplement. Annu. Rev. Nutr..

[10] Ross A.C., Caballero B., Cousins R.J., Tucker K.L., Ziegler T.R. (2012). Modern Nutrition in Health and Disease.

[11] Jin J., Mulesa L., Carrilero Rouillet M. (2017). Trace elements in parenteral nutrition: Considerations for the prescribing clinician. Nutrients.

[12] Anderson R.A. (1997). Chromium as an essential nutrient for humans. Regul. Toxicol. Pharmacol..

[13] Stearns D.M. (2000). Is chromium a trace essential metal?. Biofactors.

[14] Corradi M., Mutti A. (2011). Metal ions affecting the pulmonary and cardiovascular systems. Met. Ions Life Sci..

[15] Attenburrow M.-J., Odontiadis J., Murray B.J., Cowen P.J., Franklin M. (2002). Chromium treatment decreases the sensitivity of 5-HT2A receptors. Psychopharmacology.

[16] Piotrowska A., Młyniec K., Siwek A., Dybała M., Opoka W., Poleszak E., Nowak G. (2008). Antidepressant-like effect of chromium chloride in the mouse forced swim test: Involvement of glutamatergic and serotonergic receptors. Pharmacol. Rep..

[17] Młyniec K., Davies C.L., Gómez de Agüero Sánchez I., Pytka K., Budziszewska B., Nowak G. (2014). Essential elements in depression and anxiety. Part I. Pharmacol. Rep..

[18] Boyle E., Mondschein B., Dash H.H. (1977). Chromium depletion in the pathogenesis of diabetes and atherosclerosis. South Med. J..

[19] Khodavirdipour A., Haddadi F., Keshavarzi S. (2020). Chromium supplementation; Negotiation with diabetes mellitus, hyperlipidemia and depression. J. Diabetes Metab. Disord..

[20] Suksomboon N., Poolsup N., Yuwanakorn A. (2014). Systematic review and meta-analysis of the efficacy and safety of chromium supplementation in diabetes. J. Clin. Pharm. Ther..

[21] World Health Organization (WHO) Depression. www.who.int/news-room/fact-sheets/detail/depression.

[22] Colodro-Conde L., Couvy-Duchesne B., Zhu G., Coventry W.L., Byrne E.M., Gordon S., Wright M.J., Montgomery G.W., Madden P., Major Depressive Disorder Working Group of the Psychiatric Genomics Consortium (2018). A direct test of the diathesis-stress model for depression. Mol. Psychiatry.

[23] Lee R.D., Chen J. (2017). Adverse childhood experiences, mental health, and excessive alcohol use: Examination of race/ethnicity and sex differences. Child Abuse Negl..

[24] Zhang X., Cui L., Chen B., Xiong Q., Zhan Y., Ye J., Yin Q. (2021). Effect of chromium supplementation on hs-CRP, TNF-α and IL-6 as risk factor for cardiovascular diseases: A meta-analysis of randomized-controlled trials. Complement. Ther. Clin. Pract..

[25] Wysocka E., Cymerys M., Mielcarz G., Bryl W., Dzięgielewska S., Torliński L. (2011). The way of serum chromium utilization may contribute to cardiovascular risk factors in centrally obese persons. Arch. Med. Sci..

[26] Nasab H., Rajabi S., Eghbalian M., Malakootian M., Hashemi M., Mahmoudi-Moghaddam H. (2022). Association of As, Pb, Cr, and Zn urinary heavy metals levels with predictive indicators of cardiovascular disease and obesity in children and adolescents. Chemosphere.

[27] World Health Organization (WHO) Noncommunicable Diseases (NCDS) and Mental Health: Challenges and Solutions Infographics. www.who.int/docs/default-source/infographics-pdf/ncds/ncds-and-mental-health-sdg-in-action.pdf?sfvrsn=e1633a93_2.

[28] GBD 2019 Risk Factors Collaborators (2020). Global burden of 87 risk factors in 204 countries and territories, 1990–2019: A systematic analysis for the Global Burden of Disease Study 2019. Lancet.

[29] Roth G.A., Mensah G.A., Johnson C.O., Addolorato G., Ammirati E., Baddour L.M., Barengo N.C., Beaton A.Z., Benjamin E.J., Benziger C.P. (2020). Global burden of cardiovascular diseases and risk factors, 1990–2019: Update from the GBD 2019 study. J. Am. Coll. Cardiol..

[30] World Health Organization (WHO) Cardiovascular Diseases. www.who.int/health-topics/cardiovascular-diseases#tab=tab_1.

[31] Lin X., Xu Y., Pan X., Xu J., Ding Y., Sun X., Song X., Ren Y., Shan P.-F. (2020). Global, regional, and national burden and trend of diabetes in 195 countries and territories: An analysis from 1990 to 2025. Sci. Rep..

[32] World Health Organization (WHO) Diabetes. www.who.int/health-topics/diabetes#tab=tab_1.

[33] Tolentino J.C., Schmidt S.L. (2018). DSM-5 Criteria and depression severity: Implications for clinical practice. Front. Psychiatry.

[34] Zhou Q., Guo W., Jia Y., Xu J. (2019). Comparison of chromium and iron distribution in serum and urine among healthy people and prediabetes and diabetes patients. BioMed Res. Int..

[35] Hajra B., Orakzai B.A., Faryal U., Hassan M., Rasheed S., Wazir S. (2016). Insulin sensitivity to trace metals (chromium, manganese) in type 2 diabetic patients and non diabetic individuals. J. Ayub Med. Coll. Abbottabad.

[36] Lin C.C., Huang Y.L. (2015). Chromium, zinc and magnesium status in type 1 diabetes. Curr. Opin. Clin. Nutr. Metab. Care.

[37] Rafiei R., Habyby Z., Fouladi L., Najafi S., Asgary S., Torabi Z. (2014). Chromium level in prediction of diabetes in pre-diabetic patients. Adv. Biomed. Res..

[38] Eva H., Akter Q.S., Alam M.K. (2020). Relationship between glycemic status and serum chromium level with type 2 diabetes mellitus. Mymensingh Med. J..

[39] Chen S., Jin X., Shan Z., Li S., Yin J., Sun T., Luo C., Yang W., Yao P., Yu K. (2017). Inverse association of plasma chromium levels with newly diagnosed type 2 diabetes: A case-control study. Nutrients.

[40] Zhao F., Pan D., Wang N., Xia H., Zhang H., Wang S., Sun G. (2022). Effect of chromium supplementation on blood glucose and lipid levels in patients with type 2 diabetes mellitus: A systematic review and meta-analysis. Biol. Trace Elem. Res..

[41] Farrokhian A., Mahmoodian M., Bahmani F., Amirani E., Shafabakhsh R., Asemi Z. (2020). The influences of chromium supplementation on metabolic status in patients with type 2 diabetes mellitus and coronary heart disease. Biol. Trace Elem. Res..

[42] Simonoff M. (1984). Chromium deficiency and cardiovascular risk. Cardiovasc. Res..

[43] Alissa E.M., Bahjri S.M., Ahmed W.H., Al-Ama N., Ferns G.A.A. (2009). Chromium status and glucose tolerance in Saudi men with and without coronary artery disease. Biol. Trace Elem. Res..

[44] Ngala R.A., Awe M.A., Nsiah P. (2018). The effects of plasma chromium on lipid profile, glucose metabolism and cardiovascular risk in type 2 diabetes mellitus. A case-control study. PLoS ONE.

[45] Khan N., Hashmi S., Siddiqui A.J., Farooq S., Sami S.A., Basir N., Bokhari S.S., Sharif H., Junejo S., El-Seedi H.R. (2020). Understanding of metals dysregulation in patients with systolic and diastolic dysfunction in ischemic heart disease. Sci. Rep..

[46] Lind P.M., Olsén L., Lind L. (2012). Circulating levels of metals are related to carotid atherosclerosis in elderly. Sci. Total Environ..

[47] Xu L., Zhang S., Chen W., Yan L., Chen Y., Wen H., Liu D., Rosenblat J.D., Wang J., Cao B. (2020). Trace elements differences in the depression sensitive and resilient rat models. Biochem. Biophys. Res. Commun..

[48] Tarleton E.K., Littenberg B., MacLean C.D., Kennedy A.G., Daley C. (2017). Role of magnesium supplementation in the treatment of depression: A randomized clinical trial. PLoS ONE.

[49] Szewczyk B., Szopa A., Serefko A., Poleszak E., Nowak G. (2018). The role of magnesium and zinc in depression: Similarities and differences. Magnes. Res..

[50] Wang J., Um P., Dickerman B.A., Liu J. (2018). Zinc, magnesium, selenium and depression: A review of the evidence, potential mechanisms and implications. Nutrients.

[51] Yosaee S., Soltani S., Esteghamati A., Motevalian S.A., Tehrani-Doost M., Clark C.C.T., Jazayeri S. (2020). Effects of zinc, vitamin D, and their co-supplementation on mood, serum cortisol, and brain-derived neurotrophic factor in patients with obesity and mild to moderate depressive symptoms: A phase II, 12-wk, 2 × 2 factorial design, double-blind, randomized, placebo-controlled trial. Nutrition.

[52] Lang U.E., Beglinger C., Schweinfurth N., Walter M., Borgwardt S. (2015). Nutritional aspects of depression. Cell. Physiol. Biochem..

[53] Brownley K.A., Von Holle A., Hamer R.M., La Via M., Bulik C.M. (2013). A double-blind, randomized pilot trial of chromium picolinate for binge eating disorder: Results of the Binge Eating and Chromium (BEACh) study. J. Psychosom. Res..

[54] Center for Disease Control and Prevention (CDC)—National Center for Health Statistics (NCHS) National Health and Nutrition Examination Survey (NHANES) 2015–2016. https://wwwn.cdc.gov/nchs/nhanes/ContinuousNhanes/Default.aspx?BeginYear=2015.

[55] Chen T.C., Clark J., Riddles M.K., Mohadjer L.K., Fakhouri T.H. (2020). National Health and Nutrition Examination Survey, 2015–2018: Sample Design and Estimation Procedures.

[56] American Diabetes Association (2018). 2. Classification and diagnosis of diabetes: Standards of medical care in diabetes—2018. Diabetes Care.

[57] Little R. (2015). Laboratory Procedure Manual—Analyte: Glycohemoglobin.

[58] Center for Disease Control and Prevention (CDC)—National Center for Health Statistics (NCHS) National Health and Nutrition Examination Survey 2015–2016—Data Documentation, Codebook, and Frequencies: Glycohemoglobin. https://wwwn.cdc.gov/Nchs/Nhanes/2015-2016/GHB_I.htm.

[59] Kroenke K., Spitzer R.L. (2002). The PHQ-9: A new depression diagnostic and severity measure. Psychiatr. Ann..

[60] Kroenke K., Spitzer R.L., Williams J.B. (2001). The PHQ-9: Validity of a brief depression severity measure. J. Gen. Intern. Med..

[61] Spitzer R.L., Kroenke K., Williams J.B. (1999). Validation and utility of a self-report version of PRIME-MD: The PHQ primary care study. JAMA.

[62] Center for Disease Control and Prevention (CDC)—National Center for Health Statistics (NCHS) National Health and Nutrition Examination Survey 2015–2016—Data Documentation, Codebook, and Frequencies: Chromium & Cobalt. https://wwwn.cdc.gov/Nchs/Nhanes/2015-2016/CRCO_I.htm.

[63] Cieslak W., Pap K., Bunch D.R., Reineks E., Jackson R., Steinle R., Wang S. (2013). Highly sensitive measurement of whole blood chromium by inductively coupled plasma mass spectrometry. Clin. Biochem..

[64] Filler G., Felder S. (2014). Trace elements in dialysis. Pediatr. Nephrol..

[65] Laposata M. (2019). Laposata’s Laboratory Medicine: The Diagnosis of Disease in the Clinical Laboratory.

[66] IBM (2016). IBM SPSS Statistics for Windows.

[67] Fischbach F., Dunning M.B. (2015). A Manual of Laboratory and Diagnostic Tests.

[68] Government of Canada Canadian Guidelines for Body Weight Classification in Adults. www.canada.ca/en/health-canada/services/food-nutrition/healthy-eating/healthy-weights/canadian-guidelines-body-weight-classification-adults/questions-answers-public.html.

[69] Centers for Disease Control and Prevention (CDC) Alcohol and Public Health—Dietary Guidelines for Alcohol. https://www.cdc.gov/alcohol/fact-sheets/moderate-drinking.htm.

[70] Saltiel A.R., Kahn C.R. (2001). Insulin signalling and the regulation of glucose and lipid metabolism. Nature.

[71] Wang Z.Q., Cefalu W.T. (2010). Current concepts about chromium supplementation in type 2 diabetes and insulin resistance. Curr. Diabetes Rep..

[72] Chen G., Liu P., Pattar G.R., Tackett L., Bhonagiri P., Strawbridge A.B., Elmendorf J.S. (2006). Chromium activates glucose transporter 4 trafficking and enhances insulin-stimulated glucose transport in 3T3-L1 adipocytes via a cholesterol-dependent mechanism. Mol. Endocrinol..

[73] Ding W., Chai Z., Duan P., Feng W., Qian Q. (1998). Serum and urine chromium concentrations in elderly diabetics. Biol. Trace Elem. Res..

[74] Morris B.W., MacNeil S., Hardisty C.A., Heller S., Burgin C., Gray T.A. (1999). Chromium homeostasis in patients with type II (NIDDM) diabetes. J. Trace Elem. Med. Biol..

[75] Helton S.G., Lohoff F.W. (2015). Serotonin pathway polymorphisms and the treatment of major depressive disorder and anxiety disorders. Pharmacogenomics.

[76] Kyte B., Ifebi E., Shrestha S., Charles S., Liu F., Zhang J. (2015). High red blood cell folate is associated with an increased risk of death among adults with diabetes, a 15-year follow-up of a national cohort. Nutr. Metab. Cardiovasc. Dis..

[77] Barbash I.M., Gaglia M.A., Torguson R., Minha S., Satler L.F., Pichard A.D., Waksman R. (2013). Effect of marital status on the outcome of patients undergoing elective or urgent coronary revascularization. Am. Heart J..

[78] Consuegra-Sánchez L., Melgarejo-Moreno A., Jaulent-Huertas L., Díaz-Pastor Á., Escudero-García G., Vicente-Gilabert M., Alonso-Fernández N., Galcerá-Tomás J. (2015). Unraveling the relation between marital status and prognosis among myocardial infarction survivors: Impact of being widowed on mortality. Int. J. Cardiol..

[79] Gerward S., Tydén P., Engström G., Hedblad B. (2010). Marital status and occupation in relation to short-term case fatality after a first coronary event—A population based cohort. BMC Public Health.

[80] Hadi Khafaji H.A.R., Habib K.A., Asaad N., Singh R., Hersi A., Falaeh H.A., Saif S.A., Al-Motarreb A., Almahmeed W., Sulaiman K. (2012). Marital status and outcome of patients presenting with acute coronary syndrome: An observational report. Clin. Cardiol..

[81] Vujcic I., Vlajinac H., Dubljanin E., Vasiljevic Z., Matanovic D., Maksimovic J., Sipetic S., Marinkovic J. (2015). Long-term prognostic significance of living alone and other risk factors in patients with acute myocardial infarction. Ir. J. Med. Sci..

[82] Yokoyama H., Higuma T., Nishizaki F., Izumiyama K., Shibutani S., Yamada M., Tomita H., Abe N., Osanai T., Okumura K. (2014). Marital status and long-term mortality of male patients presenting with acute myocardial infarction. Circulation.

[83] Andersen K.K., Andersen Z.J., Olsen T.S. (2011). Predictors of early and late case-fatality in a nationwide Danish study of 26,818 patients with first-ever ischemic stroke. Stroke.

[84] Floud S., Balkwill A., Canoy D., Wright F.L., Reeves G.K., Green J., Beral V., Cairns B.J., Million Women Study Collaborators (2014). Marital status and ischemic heart disease incidence and mortality in women: A large prospective study. BMC Med..

[85] Engström G., Tydén P., Berglund G., Hansen O., Hedblad B., Janzon L. (2000). Incidence of myocardial infarction in women. A cohort study of risk factors and modifiers of effect. J. Epidemiol. Community Health.

[86] Kriegbaum M., Christensen U., Lund R., Prescott E., Osler M. (2008). Job loss and broken partnerships: Do the number of stressful life events influence the risk of ischemic heart disease in men?. Ann. Epidemiol..

[87] Ng T.P., Jin A., Chow K.Y., Feng L., Nyunt M.S.Z., Yap K.B. (2017). Age-dependent relationships between body mass index and mortality: Singapore longitudinal ageing study. PLoS ONE.

[88] Wang J.L., Schmitz N., Dewa C.S. (2010). Socioeconomic status and the risk of major depression: The Canadian National Population Health Survey. J. Epidemiol. Community Health.

[89] Levy D., Zavala-Arciniega L., Reynales-Shigematsu L.M., Fleischer N.L., Yuan Z., Li Y., Romero L.M.S., Lau Y.K., Meza R., Thrasher J.F. (2019). Measuring smoking prevalence in a middle income nation: An examination of the 100 cigarettes lifetime screen. Glob. Epidemiol..

[90] Roussel A.M., Andriollo-Sanchez M., Ferry M., Bryden N.A., Anderson R.A. (2007). Food chromium content, dietary chromium intake and related biological variables in French free-living elderly. Br. J. Nutr..

[91] Maret W. (2019). Chromium supplementation in human health, metabolic syndrome, and diabetes. Met. Ions Life Sci..

