# Supplementary material for: Blood Chromium Levels and Their Association with Cardiovascular Diseases, Diabetes, and Depression: National Health and Nutrition Examination Survey (NHANES) 2015–2016
Source: Nutrients. 2022 Jun 28;14(13):2687. doi: 10.3390/nu14132687 (PMC9268404; doi:10.3390/nu14132687)
Supplement: Supplementary file 1 [file nutrients-14-02687-s001.zip › nutrients-1777354-supplementary.pdf]

Table S1. Description of covariates.

**1. Socio-demographic Characteristics**	
**Sex**	Categories: 1) men; 2) women.
**Age**	Categories: 1) 40–49; 2) 50–59; 3) 60 years$^+$.
**Education**	Categories: 1) less than high school graduate; 2) high school graduate or some post-secondary education; 3) post-secondary graduate or above.
**Marital Status**	Categories: 1) widowed/divorced/separated; 2) married or living with partner; 3) never married.
**Family Income**	Annual family income. Categories: 1) more than $75,000 USD; 2) $65,000–$74,999 USD; 3) $55,000–$64,999 USD; 4) $45,000–$54,999 USD; 5) $35,000–$44,999 USD; 6) $25,000–$34,999 USD; 7) less than $25,000 USD.
**2. Biological Measurements**	
**Red Blood Cell Folate**	Clinical reference ranges of 317-1422 nmol/L [64] were used for red blood cell folate results. Categories: 1) Below or within range; 2) Above range. Details about sample collection and analysis are located at: www.cdc.gov/nchs/data/nhanes/nhanes_09_10/folate_f_met.pdf (accessed on 30 May 2022).
**3. Health Measures**	
**Multi-morbidity** [a]	Derived variable. Self-report of chronic condition diagnoses summed: cardiovascular diseases (congestive heart failure, coronary heart disease, angina/angina pectoris, heart attack, stroke, high blood pressure), respiratory diseases (emphysema, chronic bronchitis, chronic obstructive pulmonary disease (COPD), asthma), kidney diseases (weak/failing kidneys, kidney stones), thyroid disease, liver diseases, bone diseases (arthritis, gout), cancer. Categories: 1) no health conditions; 2) one health condition; 3) two or more health conditions.
**Diabetes Mellitus**	Based on measures of glycohemoglobin; values: ≥5.7% defined as diabetes or prediabetes [56]. Categories: 1) no; 2) yes (diabetes or prediabetes).
**Body Mass Index (BMI)**	Body mass index (BMI) categories [65] include: 1) within healthy weight range (18.5–24.9 kg/m^2); 2) overweight (≥ 25 kg/m^2 & < 30 kg/m^2); 3) obese (≥ 30 kg/m^2).
**Mental Health-related Medication** [b]	Derived variable. Ever or currently using anti-depressant, psychiatric medication(s), or medication(s) for depression, feeling worried, or anxious in the past 30 days. Categories: 1) no or not reported; 2) yes.
**Diabetes Mellitus-related Medication** [c]	Derived variable. Ever taken any mediations for diabetes in the past 30 days. Categories: 1) no; 2) yes.
**Cardiovascular Diseases-related Medication** [d]	Derived variable. Ever taken any medications for cardiovascular diseases in the past 30 days. Categories: 1) no; 2) yes.
**4. Health Behavior Variables**	
**Drinking Behavior**	Derived variable. Based on average numbers of alcoholic drinks consumed per day in the past 12 months. Categories: 1) moderate drinking (≤1 for female and ≤2 for male); 2) not moderate drinking (>1 for female and >2 for male) [66]; 3) not reported.
**Smoking**	Ever smoked 100 cigarettes or more in lifetime. Categories: 1) no; 2) yes.

[a] Cardiovascular conditions were excluded from the multi-morbidity variable for the analysis where cardiovascular disease is the dependent variable. [b] Mental health-related medication variable is only incorporated in the analysis where depression is the dependent variable. [c] Diabetes Mellitus-related medication variable is only incorporated in the analysis where diabetes mellitus is the dependent variable. [d] Cardiovascular diseases-related medication variable is only incorporated in the analysis where cardiovascular diseases is the dependent variable. 60 years$^+$ means 60 and over 60 years old. $ means American currency.

Table S2. Description of sample by health conditions (*n* = 2894 (total), 1391 (♂), 1503 (♀)).

Variable	Men			Women		
	**With Health Condition (Unweighted *n*, frequency (% of total ♂))**	**Total (Unweighted *n*, frequency (% of total ♂))**	**χ^2 (df), *p*-value**	**With Health Condition (Unweighted *n*, frequency (% of total ♀))**	**Total (Unweighted *n*, frequency (% of total ♀))**	**χ^2 (df), *p*-value**
**Cardiovascular Diseases** [a] (*n* = 723 (♂), 783 (♀))						
**1. Blood Chromium Levels**						
Within range (0.7–28.0 µg/L)	63 (4.5)	111 (8.0)	1.104 (1), 0.293	67 (4.5)	129 (8.6)	0.001 (1), 0.970
Below range (<0.7 µg/L)	660 (47.4)	1280 (92.0)		716 (47.6)	1374 (91.4)	
**2. Socio-demographic Characteristics**						
**Age**						
40–49 years	97 (7.0)	333 (23.9)	128.597 (2), 0.000	115 (7.7)	399 (26.5)	168.759 (2), 0.000
50–59 years	160 (11.5)	349 (25.1)		168 (11.2)	370 (24.6)	
60 years+	466 (33.5)	709 (51.0)		500 (33.3)	734 (48.8)	
**Education**						
Less than high school graduate	166 (11.9)	354 (25.4)	19.376 (2), 0.000	234 (15.6)	383 (25.5)	36.578 (2), 0.000
High school graduate or some post-secondary education	400 (28.8)	691 (49.7)		412 (27.4)	770 (51.2)	
Post-secondary graduate or above	157 (11.3)	346 (24.9)		137 (9.1)	350 (23.3)	
**Marital Status**						
Widowed or divorced or separated	165 (11.9)	273 (19.6)	9.889 (2), 0.007	331 (22.0)	544 (36.2)	29.189 (2), 0.000
Married or living with partner	504 (36.2)	1006 (72.3)		378 (25.1)	822 (54.7)	
Never married	54 (3.9)	112 (8.1)		74 (4.9)	137 (9.1)	
**Family Income**						
$75,000+ USD	171 (12.3)	368 (26.5)	25.337 (7), 0.001	133 (8.8)	351 (23.4)	75.535 (7), 0.000
$65,000–$74,999 USD	29 (2.1)	70 (5.0)		24 (1.6)	63 (4.2)	

$55,000–$64,999 USD	40 (2.9)	87 (6.3)		44 (2.9)	94 (6.3)	
$45,000–$54,999 USD	55 (4.0)	101 (7.3)		66 (4.4)	116 (7.7)	
$35,000–$44,999 USD	64 (4.6)	125 (9.0)		58 (3.9)	122 (8.1)	
$25,000–$34,999 USD	86 (6.2)	183 (13.2)		124 (8.3)	227 (15.1)	
<$25,000 USD	250 (18.0)	410 (29.5)		311 (20.7)	473 (31.5)	
Not reported	28 (2.0)	47 (3.4)		23 (1.5)	57 (3.8)	
**3. Biological Measurements**						
**Red Blood Cell Folate** [b]						
Below/within range (<317 or 317-1422 nmol/L)	475 (34.1)	996 (71.6)	25.815 (1), 0.000	506 (33.7)	1038 (69.1)	15.071 (1), 0.000
Above range (>1422 nmol/L)	248 (17.8)	395 (28.4)		277 (18.4)	465 (30.9)	
**4. Health Measures**						
**Multi-morbidity** [c]						
No health conditions	216 (15.5)	614 (44.1)		188 (12.5)	544 (36.2)	
One health condition	208 (15.0)	378 (27.2)	155.086 (2), 0.000	257 (17.1)	467 (31.1)	123.001 (2), 0.000
Two or more health conditions	299 (21.5)	399 (28.7)		338 (22.5)	492 (32.7)	
**Diabetes Mellitus (DM)**						
Yes (glycohemoglobin ≥5.7%)	477 (34.3)	783 (56.3)	57.391 (1), 0.000	529 (35.2)	829 (55.2)	101.680 (1), 0.000
No (glycohemoglobin <5.7%)	246 (17.7)	608 (43.7)		254 (16.9)	674 (44.8)	
**Depression** [d]						
Yes	168 (12.1)	296 (21.3)	3.442 (1), 0.064	279 (18.6)	445 (29.6)	28.467 (1), 0.000
No	555 (39.9)	1095 (78.7)		504 (33.5)	1058 (70.4)	
**Body Mass Index (BMI)** [e]						
Within healthy weight range	121 (8.7)	308 (22.1)		119 (7.9)	341 (22.7)	
Overweight	277 (19.9)	559 (40.2)	42.377 (2), 0.000	203 (13.5)	419 (27.9)	72.125 (2), 0.000
Obese	325 (23.4)	524 (37.7)		461 (30.7)	743 (49.4)	
**Taking Cardiovascular Disease-related Medication**						
Yes	595 (42.8)	726 (52.2)	546.781 (1), 0.000	655 (43.6)	753 (50.1)	736.051 (1), 0.000
No	128 (9.2)	665 (47.8)		128 (8.5)	750 (49.9)	
**5. Health Behavior Variables**						
**Drinking Behavior** [f]						

Moderate drinking	301 (21.6)	580 (41.7)	5.628 (2), 0.060	210 (14.0)	431 (28.7)	23.204 (2), 0.000
Not moderate drinking	184 (13.2)	386 (27.7)		177 (11.8)	398 (26.5)	
Not reported	238 (17.1)	425 (30.6)		396 (26.3)	674 (44.8)	
**Smoking** [g]						
< 100 cigarettes	251 (18.0)	571 (41.0)	24.954 (1), 0.000	492 (32.7)	986 (65.6)	5.545 (1), 0.019
≥ 100 cigarettes	472 (33.9)	820 (59.0)		291 (19.4)	517 (34.4)	
**Diabetes Mellitus** [h] (n = 783 (♂), 829 (♀))						
**1. Blood Chromium Levels**						
Within range (0.7–28.0 μg/L)	65 (4.7)	111 (8.0)	0.252 (1), 0.616	77 (5.1)	129 (8.6)	1.173 (1), 0.279
Below range (<0.7 μg/L)	718 (51.6)	1280 (92.0)		752 (50.0)	1374 (91.4)	
**2. Socio-demographic Characteristics**						
**Age**						
40–49 years	131 (9.4)	333 (23.9)	59.597 (2), 0.000	145 (9.6)	399 (26.5)	88.219 (2), 0.000
50–59 years	193 (13.9)	349 (25.1)		204 (13.6)	370 (24.6)	
60 years+	459 (33.0)	709 (51.0)		480 (31.9)	734 (48.8)	
**Education**						
Less than high school graduate	220 (15.8)	354 (25.4)	12.235 (2), 0.002	253 (16.8)	383 (25.5)	26.374 (2), 0.000
High school graduate or some secondary education	393 (28.3)	691 (49.7)		406 (27.0)	770 (51.2)	
Post-secondary graduate or above	170 (12.2)	346 (24.9)		170 (11.3)	350 (23.3)	
**Marital Status**						
Widowed or divorced or separated	153 (11.0)	273 (19.6)	3.357 (2), 0.187	326 (21.7)	544 (36.2)	8.754 (2), 0.013
Married or living with partner	576 (41.4)	1006 (72.3)		426 (28.3)	822 (54.7)	
Never married	54 (3.9)	112 (8.1)		77 (5.1)	137 (9.1)	
**Family Income**						
\$75,000+ USD	183 (13.2)	368 (26.5)	14.479 (7), 0.043	147 (9.8)	351 (23.4)	53.828 (7), 0.000
\$65,000–\$74,999 USD	39 (2.8)	70 (5.0)		30 (2.0)	63 (4.2)	
\$55,000–\$64,999 USD	47 (3.4)	87 (6.3)		47 (3.1)	94 (6.3)	
\$45,000–\$54,999 USD	61 (4.4)	101 (7.3)		63 (4.2)	116 (7.7)	
\$35,000–\$44,999 USD	81 (5.8)	125 (9.0)		62 (4.1)	122 (8.1)	

$25,000–$34,999 USD	99 (7.1)	183 (13.2)		132 (8.8)	227 (15.1)	
<$25,000 USD	248 (17.8)	410 (29.5)		314 (20.9)	473 (31.5)	
Not reported	25 (1.8)	47 (3.4)		34 (2.3)	57 (3.8)	
**3. Biological Measurements**						
**Red Blood Cell Folate [b]**						
Below/within range (<317 or 317–1422 nmol/L)	551 (39.6)	996 (71.6)	1.339 (1),	558 (37.1)	1038 (69.1)	2.655 (1),
Above range (>1422 nmol/L)	232 (16.7)	395 (28.4)	0.247	271 (18.0)	465 (30.9)	0.103
**4. Health Measures**						
**Multi-morbidity [i]**						
No health conditions	174 (12.5)	398 (28.6)		142 (9.4)	356 (23.7)	
One health condition	199 (14.3)	349 (25.1)	39.875 (2),	192 (12.8)	378 (25.1)	62.853 (2),
Two or more health conditions	410 (29.5)	644 (46.3)	0.000	495 (32.9)	769 (51.2)	0.000
**Depression [d]**						
Yes	181 (13.0)	296 (21.3)	3.607 (1),	273 (18.2)	445 (29.6)	9.799 (1),
No	602 (43.3)	1095 (78.7)	0.058	556 (37.0)	1058 (70.4)	0.002
**BMI [e]**						
Within healthy weight range	139 (10.0)	308 (22.1)	39.944 (2),	114 (7.6)	341 (22.7)	113.142 (2),
Overweight	296 (21.3)	559 (40.2)	0.000	214 (14.2)	419 (27.9)	0.000
Obese	348 (25.0)	524 (37.7)		501 (33.3)	743 (49.4)	
**Taking Diabetes Mellitus-related Medication**						
Yes	271 (19.5)	283 (20.3)	224.947 (1),	251 (16.7)	264 (17.6)	206.330 (1),
No	512 (36.8)	1108 (79.7)	0.000	578 (38.5)	1239 (82.4)	0.000
**5. Health Behavior Variables**						
**Drinking Behavior [f]**						
Moderate drinking	322 (23.1)	580 (41.7)	17.627 (2),	227 (15.1)	431 (28.7)	47.764 (2),
Not moderate drinking	190 (13.7)	386 (27.7)	0.000	170 (11.3)	398 (26.5)	0.000
Not reported	271 (19.5)	425 (30.6)		432 (28.7)	674 (44.8)	
**Smoking [g]**						
<100 cigarettes	309 (22.2)	571 (41.0)	1.862 (1),	545 (36.3)	986 (65.6)	0.016 (1),
≥100 cigarettes	474 (34.1)	820 (59.0)	0.172	284 (18.9)	517 (34.4)	0.899
			**Depression [d]** (*n* = 296 (♂), 445 (♀))			
**1. Blood Chromium Levels**						

Within range (0.7–28.0 μg/L)	23 (1.7)	111 (8.0)	0.022 (1),	36 (2.4)	129 (8.6)	0.196 (1),
Below range (< 0.7 μg/L)	273 (19.6)	1280 (92.0)	0.881	409 (27.2)	1374 (91.4)	0.658
**2. Socio-demographic Characteristics**						
**Age**						
40–49 years	53 (3.8)	333 (23.9)	15.645 (2), 0.000	115 (7.7)	399 (26.5)	0.960 (2), 0.619
50–59 years	98 (7.0)	349 (25.1)		117 (7.8)	370 (24.6)	
60 years+	145 (10.4)	709 (51.0)		213 (14.2)	734 (48.8)	
**Education**						
Less than high school graduate	104 (7.5)	354 (25.4)	23.713 (2), 0.000	144 (9.6)	383 (25.5)	25.451 (2), 0.000
High school graduate or some secondary education	142 (10.2)	691 (49.7)		229 (15.2)	770 (51.2)	
Post-secondary graduate or above	50 (3.6)	346 (24.9)		72 (4.8)	350 (23.3)	
**Marital Status**						
Widowed or divorced or separated	78 (5.6)	273 (19.6)	14.726 (2), 0.001	181 (12.0)	544 (36.2)	7.108 (2), 0.029
Married or living with partner	188 (13.5)	1006 (72.3)		220 (14.6)	822 (54.7)	
Never married	30 (2.2)	112 (8.1)		44 (2.9)	137 (9.1)	
**Family Income**						
$75,000+ USD	40 (2.9)	368 (26.5)	51.223 (7), 0.000	64 (4.3)	351 (23.4)	51.247 (7), 0.000
$65,000–$74,999 USD	10 (0.7)	70 (5.0)		15 (1.0)	63 (4.2)	
$55,000–$64,999 USD	16 (1.2)	87 (6.3)		27 (1.8)	94 (6.3)	
$45,000–$54,999 USD	24 (1.7)	101 (7.3)		31 (2.1)	116 (7.7)	
$35,000–$44,999 USD	22 (1.6)	125 (9.0)		34 (2.3)	122 (8.1)	
$25,000–$34,999 USD	48 (3.5)	183 (13.2)		70 (4.7)	227 (15.1)	
<$25,000 USD	125 (9.0)	410 (29.5)		191 (12.7)	473 (31.5)	
Not reported	11 (0.8)	47 (3.4)		13 (0.9)	57 (3.8)	
**3. Biological Measurements**						
**Red Blood Cell Folate** [b]						
Below/within range (<317 or 317–1422 nmol/L)	210 (15.1)	996 (71.6)	0.080 (1), 0.777	297 (19.8)	1038 (69.1)	1.593 (1), 0.207
Above range (>1422 nmol/L)	86 (6.2)	395 (28.4)		148 (9.8)	465 (30.9)	

**4. Health Measures**						
**Multi-morbidity [i]**						
No health conditions	59 (4.2)	398 (28.6)		61 (4.1)	356 (23.7)	
One health condition	56 (4.0)	349 (25.1)	33.521 (2), 0.000	84 (5.6)	378 (25.1)	69.097 (2), 0.000
Two or more health conditions	181 (13.0)	644 (46.3)		300 (20.0)	769 (51.2)	
**Diabetes Mellitus**						
Yes (glycohemoglobin ≥5.7%)	181 (13.0)	783 (56.3)	3.607 (1), 0.058	273 (18.2)	829 (55.2)	9.799 (1), 0.002
No (glycohemoglobin <5.7%)	115 (8.3)	608 (43.7)		172 (11.4)	674 (44.8)	
**BMI [e]**						
Within healthy weight range	62 (4.5)	308 (22.1)		75 (5.0)	341 (22.7)	
Overweight	105 (7.5)	559 (40.2)	5.809 (2), 0.055	113 (7.5)	419 (27.9)	19.732 (2), 0.000
Obese	129 (9.3)	524 (37.7)		257 (17.1)	743 (49.4)	
**Taking Mental Health-related Medication [j]**						
Yes	99 (7.1)	209 (15.0)	99.934 (1), 0.000	195 (13.0)	361 (24.0)	135.825 (1), 0.000
No or not reported	197 (14.2)	1182 (85.0)		250 (16.6)	1142 (76.0)	
**5. Health Behavior Variables**						
**Drinking Behavior [f]**						
Moderate drinking	101 (7.3)	580 (41.7)		108 (7.2)	431 (28.7)	
Not moderate drinking	100 (7.2)	386 (27.7)	10.400 (2), 0.006	129 (8.6)	398 (26.5)	6.290 (2), 0.043
Not reported	95 (6.8)	425 (30.6)		208 (13.8)	674 (44.8)	
**Smoking [g]**						
<100 cigarettes	93 (6.7)	571 (41.0)	14.412 (1), 0.000	250 (16.6)	986 (65.6)	24.872 (1), 0.000
≥100 cigarettes	203 (14.6)	820 (59.0)		195 (13.0)	517 (34.4)	

[a] Based on self report of congestive heart failure, coronary heart disease, angina/angina pectoris, heart attack, stroke, or hypertension. [b] Clinical reference ranges of 317-1422 nmol/L [67]. [c] Includes respiratory diseases (emphysema, chronic bronchitis, chronic obstructive pulmonary disease (COPD), asthma), kidney diseases (weak/failing kidneys, kidney stones), thyroid disease, liver diseases, bone diseases (arthritis, gout), cancer. [d] Depression: total Patient Health Questionnaire-9 (PHQ-9) score ≥5 [59,60]. [e] BMI: body mass index. Within healthy weight range: 18.5-24.9 kg/m^2; overweight: 25-30 kg/m^2; obese: ≥30 kg/m^2 [68]. [f] Moderate drinking: ≤1 alcoholic drinks/day for female and ≤2 alcoholic drinks/day for male. Not moderate drinking: >1 alcoholic drinks/day for women and >2 alcoholic drinks/day for men [69]. [g] Number of cigarettes smoked in lifetime. [h] Glycohemoglobin <5.7% (no prediabetes or diabetes; code 0) and ≥5.7% (prediabetes or diabetes; code 1). [i] Includes cardiovascular diseases (CVDs) (congestive heart failure, coronary heart disease, angina/angina pectoris, heart attack, stroke, high blood pressure), respiratory diseases

(emphysema, chronic bronchitis, chronic obstructive pulmonary disease (COPD), asthma), kidney diseases (weak/failing kidneys, kidney stones), thyroid disease, liver diseases, bone diseases (arthritis, gout), cancer. [j] Includes anti-depressant, psychiatric medication(s), or medication(s) for depression, feeling worried, or anxious. $75{,}000^{+}$ USD means 75,000 and over 75,000 USD. 60 years^{+} means 60 and over 60 years old. $ means American currency. ♂, male; ♀, female.
